# Postoperative insulin resistance and the intestinal microbiota: mechanisms and research advances

**DOI:** 10.20517/mrr.2025.54

**Published:** 2025-09-25

**Authors:** Feng Lin, Minzhi Sun, Xiao Yuan, Yujie Cai, Wenjing Chen, Siyu Liu, Zhipeng He

**Affiliations:** ^1^Department of General Surgery, The First Affiliated Hospital of Anhui Medical University. Hefei 230022, Anhui, China.; ^2^Anhui Public Health Clinical Center. Hefei 230032, Anhui, China.; ^3^School of Pharmacy, Jiangxi Medical College, Nanchang University, Nanchang 330031, Jiangxi, China.; ^4^Department of Radiology, Zhejiang Cancer Hospital, Hangzhou Institute of Medicine (HIM), Chinese Academy of Sciences, Hangzhou 310000, Zhejiang, China.; ^5^Department of General Surgery, The First Affiliated Hospital of Zhejiang Chinese Medical University (Zhejiang Provincial Hospital of Chinese Medicine), Hangzhou 310000, Zhejiang, China.; ^#^The authors have contributed equally to the work.

**Keywords:** Postoperative insulin resistance, gut microbiota, metabolic products, postoperative recovery, short-chain fatty acids

## Abstract

Postoperative insulin resistance (PIR) is a common metabolic complication that significantly affects patient recovery and long-term outcomes. Recent studies have revealed a robust association between the gut microbiota and PIR, underscoring the potential role of microbial communities in modulating insulin sensitivity. In this comprehensive review, we synthesize current literature on the interplay between PIR and the gut microbiota, delve into the underlying mechanisms linking the two, and provide an overview of recent research progress in this field. Evidence suggests that the gut microbiota may influence PIR through mechanisms involving metabolic endotoxins, short-chain fatty acids, branched-chain amino acids, and other metabolites. Overall, the gut microbiota plays a crucial role in the onset and progression of PIR. This review aims to provide a theoretical basis for developing PIR intervention strategies based on microbiome regulation.

## INTRODUCTION

Postoperative insulin resistance (PIR) refers to a reduction in insulin sensitivity that occurs after surgery. This metabolic complication not only impairs postoperative recovery but may also increase the risk of infections, delayed wound healing, and other adverse outcomes^[1-3]^. The pathogenesis of PIR is multifactorial, involving the surgical stress response, inflammatory reactions, and metabolic disturbances. In recent years, growing evidence has suggested that the gut microbiota also plays a critical role in the development of PIR. Table 1 shows some studies on the association between gut microbiota and PIR^[4-10]^. For the assessment of PIR, the Homeostasis Model Assessment (HOMA) remains the most commonly employed method for estimating the insulin resistance index (HOMA-IR). This approach requires only fasting plasma glucose (FPG, mmol/L) and fasting insulin (FINS, mU/ml) levels to evaluate both the degree of insulin resistance and residual β-cell function. The HOMA-IR value is calculated using the formula: HOMA-IR = [FPG (mmol/L) × FINS (mU/ml)] / 22.5^[5]^.

**Table 1:** Studies on the association between gut microbiota and PIR

**References**	**Country**	**Year**	**Type of surgery**	**Gut microbiota examined**	**Research subject**	**Evaluation items**
He *et al.*^[4]^	China	2024	Gastrectomy	*Bifidobacterium, Faecalibacterium*	Human	PIR; Inflammation markers; Nutrition indicators
Liu *et al.*^[5]^	China	2023	Gastrectomy	*Faecalibacterium prausnitzii; Gemmiger formicilis*	Human	PIR; Inflammation markers; Infectious complications
Prudêncio *et al.*^[6]^	Brazil	2023	Roux-en-Y Gastric bypass	*Dorea longicatena*	Human	PIR
Shi *et al.*^[7]^	China	2021	Roux-en-Y Gastric Bypass	*Akkemansia muciniphila*	Human	PIR
Basso *et al.*^[8]^	USA	2017	Sleeve gastrectomy	*Firmicutes; Actinobacteria*	Rat	PIR
Jahansouz *et al.*^[9]^	Italy	2016	Vertical sleeve gastrectomy	*Ruminococcus; Lactobacillus; Collinsella*	Rat	PIR

PIR: Postoperative insulin resistance.

The gut microbiota refers to the microbial community in the human intestine, including bacteria, viruses, fungi, and archaea. It is vast in quantity and highly diverse in species, with a total genomic content that far exceeds that of the human genome, earning it the designation of the “second genome”^[11]^. The gut microbiota influences the host’s metabolic state through multiple pathways, such as metabolite production and immune regulation, and participates in numerous physiological processes, including energy balance, nutrient absorption, immune modulation, and inflammatory responses^[12]^. In recent years, increasing evidence has indicated that the composition and function of the gut microbiota are closely associated with PIR^[13,14]^. Gut dysbiosis is considered a key pathogenic factor in PIR. Dysbiosis can impair intestinal barrier function and increase intestinal permeability, allowing bacterial endotoxins such as lipopolysaccharide (LPS) to enter the bloodstream, triggering systemic inflammatory responses, interfering with insulin signaling pathways, and ultimately causing insulin resistance^[15]^. Furthermore, gut-derived metabolites such as short-chain fatty acids (SCFAs) and branched-chain amino acids (BCAAs) also play significant roles in the development of PIR^[16,17]^. SCFAs, the main products of dietary fiber fermentation by gut microbes, exert anti-inflammatory effects and regulate energy metabolism^[18]^. Reduced SCFA levels have been linked to PIR, whereas elevated BCAA levels are strongly associated with impaired insulin sensitivity.

**Figure 1:**
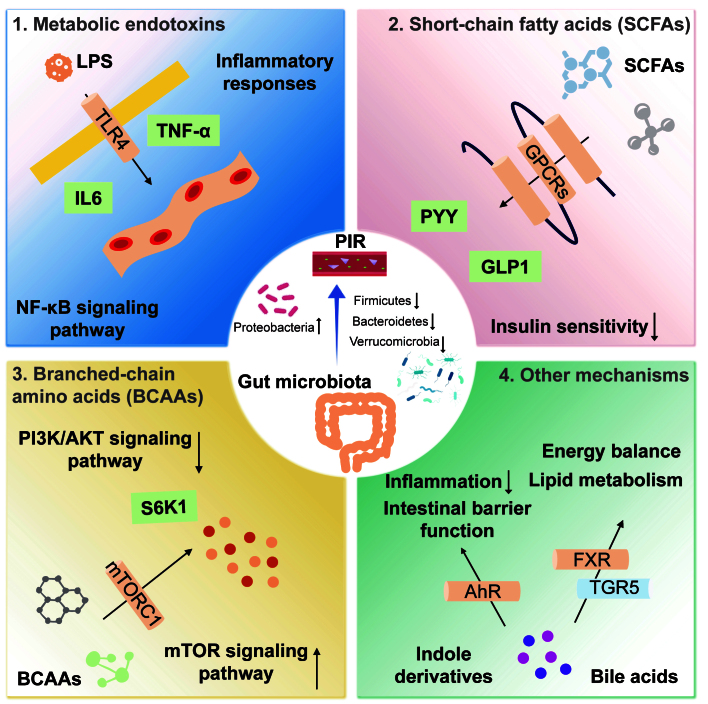
Mechanisms by which the gut microbiota influences postoperative insulin resistance. (1) LPS is a potent inflammatory mediator that binds to TLR4, activating the NF-κB signaling pathway and inducing the release of inflammatory cytokines, such as TNF-α and IL-6; (2) SCFAs act via GPCRs, whose activation promotes the secretion of GLP-1 and PYY; (3) BCAAs impair insulin signaling by inhibiting the PI3K/AKT pathway through activation of the mTORC1. mTORC1 activation induces phosphorylation of downstream S6K1; (4) Bile acids enhance insulin sensitivity by activating receptors such as the FXR and TGR5. Indole derivatives regulate host inflammation and metabolic health by activating the AhR. AhR activation alleviates inflammation, improves intestinal barrier function, and may enhance insulin sensitivity by modulating metabolic pathways. LPS: Lipopolysaccharide; TLR4: Toll-like receptor 4; NF-κB: nuclear factor-κB; TNF-α: Tumor necrosis factor-α; IL-6: interleukin-6; SCFA: short-chain fatty acid; GPCR: G protein-coupled receptor; GLP-1: glucagon-like peptide-1; PYY: peptide YY; BCAA: branched-chain amino acid; mTORC1: mammalian target of rapamycin complex 1; PI3K: phosphatidylinositol 3-kinase; protein kinase B; FXR: farnesoid X receptor; TGR5: G protein-coupled receptor 5; AhR: aryl hydrocarbon receptor; PIR: postoperative insulin resistance.

Given the critical role of the gut microbiota in PIR, modulating its structure and function has emerged as a novel strategy for prevention and treatment. Interventions such as probiotics, prebiotics, and fecal microbiota transplantation (FMT) show promising potential in ameliorating PIR. This review discusses the relationship between PIR and the gut microbiota, explores the underlying mechanisms, and summarizes recent research progress. It also provides new insights and references for PIR research and clinical management [Figure 1].

## MECHANISMS UNDERLYING POSTOPERATIVE INSULIN RESISTANCE

### Surgical stress response

The surgical stress response is a key contributor to PIR. During surgery, tissue injury and physiological stress trigger a cascade of complex biological reactions^[19,20]^. In this process, the massive release of stress hormones, such as epinephrine and cortisol, profoundly disrupts the body’s metabolic balance. These hormones reduce insulin sensitivity through several molecular mechanisms, including the inhibition of critical components in the insulin signal transduction pathway^[21,22]^. Specifically, stress hormones stimulate hepatic gluconeogenesis, promoting the conversion of noncarbohydrate substrates into glucose, while also accelerating glycogenolysis. Together, these processes elevate blood glucose levels^[23]^. Although this metabolic shift provides an immediate energy supply, it simultaneously disrupts glucose homeostasis. In parallel, the surgical stress response induces a systemic inflammatory reaction. Inflammatory mediators, such as cytokines and acute-phase proteins, are released into circulation, further impairing insulin signaling and exacerbating insulin resistance^[24,25]^. This interplay between inflammation and insulin resistance constitutes a complex regulatory network during postoperative recovery. A deeper understanding of the surgical stress response and its impact on insulin sensitivity is crucial for optimizing clinical management strategies and improving patient outcomes.

### Inflammatory response

Following surgery, the body mounts a rapid response mechanism known as the acute inflammatory response. While this reaction is protective against surgical trauma, it is also characterized by the release of large amounts of inflammatory mediators. Tumor necrosis factor-α (TNF-α) and interleukin-6 (IL-6) are representative cytokines that play pivotal roles in immune regulation and inflammatory processes^[26-28]^. These cytokines act not only at the site of injury but also disseminate via the bloodstream to affect distant organs. By activating the nuclear factor-κB (NF-κB) signaling pathway, TNF-α and IL-6 influence the expression of numerous genes, including those involved in insulin signaling. Activation of this pathway induces both pro- and anti-inflammatory gene expression, ultimately suppressing insulin signal transduction and reducing insulin sensitivity^[28,29]^.

Moreover, the inflammatory response can compromise the integrity of the intestinal mucosal barrier, increasing permeability and allowing bacterial endotoxins such as LPS to translocate into the bloodstream^[30,31]^. Circulating LPS further stimulates systemic inflammation and binds to Toll-like receptor 4 (TLR4) on immune cells, amplifying insulin resistance. Clinical studies indicate that the intensity of the postoperative inflammatory response is closely correlated with PIR severity^[32,33]^. Therefore, controlling the inflammatory response may help alleviate PIR and improve metabolic stability and recovery. Further investigations into the specific roles of inflammatory cytokines, as well as strategies to modulate intestinal barrier function and reduce LPS-induced inflammation, are warranted. Such insights could provide clues for the development of novel therapeutic strategies to improve patient rehabilitation.

## RELATIONSHIP BETWEEN THE GUT MICROBIOTA AND PIR

### Composition and diversity of the gut microbiota

The development of PIR is a complex pathological process influenced by multiple factors, among which the composition and diversity of the gut microbiota play crucial roles. Recent research has revealed a close link between the gut microbiota and PIR, particularly regarding changes in microbial diversity and the abundance of specific taxa^[34]^. In patients with PIR, gut microbial diversity is generally reduced, reflecting an imbalance in the structure of the intestinal microbial community. The ratio of Firmicutes to Bacteroidetes, the two dominant phyla in the gut microbiota, has been shown to correlate closely with host insulin sensitivity^[5]^. Specifically, PIR patients often exhibit an increased proportion of Firmicutes and a decreased proportion of Bacteroidetes. This shift in microbial composition may contribute to insulin resistance by affecting energy intake and storage^[35]^.

Perioperative antibiotic use is another significant factor that can induce gut dysbiosis. Antibiotic therapy not only diminishes gut microbial diversity but also reduces the abundance of beneficial bacteria such as *Bifidobacterium* and *Lactobacillus*. A reduction in these protective microbes may compromise intestinal barrier function and increase gut permeability, allowing bacterial endotoxins such as LPS to enter the bloodstream, triggering systemic inflammation and thereby increasing the risk of PIR. Several studies have confirmed the link between antibiotic-induced gut dysbiosis and PIR development^[36-38]^. Understanding the interactions between the gut microbiota and PIR is therefore vital for designing new preventive and therapeutic strategies.

### Role of gut microbiota metabolites

The metabolic activity of the gut microbiota profoundly impacts host metabolism, particularly in the context of PIR. Short-chain fatty acids (SCFAs) - including acetate, propionate, and butyrate - are primary metabolites produced by the fermentation of dietary fiber. SCFAs play key roles in regulating host metabolism and immune function. Evidence indicates that decreased SCFA levels are closely associated with PIR^[39]^. SCFAs stimulate the secretion of TLR (GLP-1) and peptide YY (PYY) from intestinal L cells, hormones that enhance insulin secretion and improve insulin sensitivity. Additionally, butyrate, a major energy source for intestinal epithelial cells, exhibits strong anti-inflammatory effects, helps maintain the integrity of the intestinal mucosal barrier, and mitigates systemic inflammation^[40]^.

Beyond SCFAs, BCAAs are also important gut microbiota-derived metabolites. Elevated BCAA levels are strongly associated with PIR, potentially contributing to insulin resistance by affecting insulin signaling and promoting muscle protein synthesis. In summary, both the composition and function of the gut microbiota, along with its metabolites, play critical roles in the onset and progression of PIR^[41]^. Factors such as perioperative antibiotic use, reduced microbial diversity, and alterations in specific metabolite levels may affect insulin sensitivity through multiple mechanisms. Future research should delve deeper into the complex relationship between the gut microbiota and PIR, elucidate underlying molecular mechanisms, and develop effective interventions to improve postoperative metabolic health in patients.

## MECHANISMS BY WHICH THE GUT MICROBIOTA INFLUENCES POSTOPERATIVE INSULIN RESISTANCE

PIR is a common metabolic complication that can adversely affect patient recovery and prognosis. Increasing evidence in recent years indicates that the gut microbiota plays a key role in the occurrence and development of PIR. The following section details the mechanisms through which the gut microbiota influences PIR.

### Metabolic endotoxins

Metabolic endotoxemia is characterized by elevated circulating endotoxins, such as LPS, resulting from an imbalance in the intestinal microbiota. This condition is associated with impaired intestinal mucosal barrier function, leading to increased intestinal permeability. The enhanced permeability allows bacterial endotoxins, particularly LPS, to cross the intestinal wall into the bloodstream, initiating a cascade of inflammatory responses. LPS, a potent inflammatory stimulus, binds to TLR4 and activates the NF-κB signaling pathway^[42,43]^. This activation induces the production of proinflammatory cytokines, including TNF-α and IL-6. These cytokines not only mediate local immune responses but also circulate systemically, further impairing insulin signaling and contributing to insulin resistance^[43,44]^. The inhibitory effects of inflammatory cytokines on insulin signaling occur through multiple mechanisms. For example, TNF-α promotes the phosphorylation of insulin receptor substrate (IRS)-1, blocking its binding to the insulin receptor and thereby inhibiting insulin signal transmission.

IL-6, in turn, activates suppressor of cytokine signaling (SOCS) proteins, further impeding key molecules in the insulin signaling pathway, exacerbating insulin resistance. The association between metabolic endotoxemia and PIR underscores the importance of maintaining gut health for systemic metabolic balance^[45]^. Further research is warranted to clarify how gut dysbiosis and metabolic endotoxemia interact and collectively influence the metabolic status of postoperative patients. A deeper understanding of these mechanisms could provide a foundation for developing new therapeutic strategies to mitigate metabolic complications in his population.

### Metabolite-mediated PIR mechanism

Surgery is a potent physiological stressor that triggers a series of complex metabolic changes in the body. While these changes are essential for recovery, they can also negatively affect insulin sensitivity. Under the combined influence of the surgical stress response and the subsequent inflammatory reaction, metabolic pathways undergo significant shifts^[46,47]^. First, gluconeogenesis is activated to meet urgent energy demands^[48]^, converting noncarbohydrate substrates into glucose and thereby increasing blood glucose levels. Concurrently, glycogen synthesis is suppressed, reducing the body’s capacity to regulate glucose effectively. Lipolysis in adipose tissue is also enhanced, releasing free fatty acids into the bloodstream. While this provides an alternative energy source, it may interfere with insulin signal transduction. These metabolic alterations substantially impact the insulin signaling pathway^[49,50]^. Key signaling molecules, such as IRS and phosphatidylinositol 3-kinase (PI3K), play central roles in transmitting insulin signals. Postoperatively, changes in the expression and activity of these molecules can hinder insulin signaling, exacerbating insulin resistance^[51,52]^.

Furthermore, the increased energy demands after surgery can further influence metabolic pathways and insulin sensitivity. For example, enhanced fatty acid oxidation to meet higher energy expenditure may also inhibit insulin signaling. The complexity and diversity of postoperative metabolic changes present considerable challenges for the management and treatment of PIR^[36,53]^. Addressing this issue requires a deeper understanding of surgery-induced metabolic alterations and the development of targeted therapeutic strategies.

SCFAs, including acetate, propionate, and butyrate, are primary metabolites produced by the fermentation of dietary fiber by gut microbiota and significantly impact host metabolic health. SCFAs play important roles in regulating insulin sensitivity and maintaining metabolic homeostasis, and reduced SCFA levels are closely linked to the occurrence of PIR^[42,54]^. SCFAs exert their effects by binding to G protein-coupled receptors (GPCRs), such as GPR41 and GPR43. Activation of these receptors stimulates intestinal L cells to secrete GLP-1 and PYY, gut hormones known to increase insulin secretion and improve insulin sensitivity. Reports indicate that GLP 43 is coupled to the Gq or Gi/o signaling pathways, while GLP 41 couples only to Gi^[55]^. Butyrate, the main energy source for intestinal epithelial cells, also possesses potent anti-inflammatory properties^[56]^. It maintains intestinal barrier integrity, reduces the translocation of bacterial endotoxins such as LPS, and consequently diminishes systemic inflammation.

SCFAs further promote insulin secretion and enhance insulin sensitivity by regulating gut hormone release. These mechanisms underscore the importance of SCFAs in postoperative metabolic recovery. However, perioperative stress responses and antibiotic use can reduce SCFA levels, affecting the interaction between the gut microbiota and host metabolism^[57,58]^. Maintaining gut microbiota balance and adequate SCFA levels may therefore have clinical significance for preventing and treating PIR.

BCAAs, including leucine, isoleucine, and valine, are essential amino acids that play key roles in metabolism and immune regulation^[59]^. Recent research has shown that elevated BCAA levels are closely associated with PIR. The gut microbiota can metabolize BCAAs, influencing host insulin signaling pathways and reducing insulin sensitivity. Mechanistically, BCAAs inhibit the PI3K/protein kinase B (AKT) pathway by activating the mammalian target of rapamycin complex 1 (mTORC1)^[60,61]^. Activation of mTORC1 induces phosphorylation of the downstream kinase S6K1, which in turn mediates phosphorylation of IRS1 at S636/S639, accelerating IRS-1 degradation and impairing insulin signal transduction^[59]^.

Moreover, BCAA metabolites, such as branched-chain fatty acids (BCFAs), may exacerbate insulin resistance by disrupting mitochondrial function and energy metabolism. BCFA accumulation can impair mitochondrial oxidative phosphorylation, causing energy metabolism imbalances that further affect insulin signaling^[62,63]^. The elevation of BCAA levels may reflect the metabolic activities of the gut microbiota, highlighting complex interactions between the gut microbiota and host metabolism.

### Other mechanisms

In addition to the well-established effects of metabolic endotoxemia, SCFAs, and BCAAs, the gut microbiota influences postoperative insulin resistance through a range of other metabolites and signaling pathways. The diversity and complexity of these mechanisms further underscore the close connection between the gut microbiota and host metabolism.

Bile acids are important metabolites produced by gut microbiota. By activating receptors such as the farnesoid X receptor (FXR) and G protein-coupled receptor 5 (TGR5), bile acids regulate lipid metabolism and energy homeostasis^[64,65]^. Activation of these receptors has been shown to improve insulin sensitivity, indicating that bile acids may play a positive role in regulating host metabolism and insulin signaling. Indole derivatives, generated from tryptophan metabolism by the gut microbiota, are also key regulators of host inflammatory responses and metabolic health through activation of the aryl hydrocarbon receptor (AhR)^[66,67]^. AhR activation can mitigate inflammation, strengthen intestinal barrier function, and potentially improve insulin sensitivity by influencing metabolic pathways.

Collectively, these findings suggest that gut microbiota-derived metabolites interact with host metabolic processes through multiple signaling pathways, influencing the onset and progression of PIR. Metabolic endotoxemia, SCFA fluctuations, BCAA metabolic disturbances, and the actions of other microbial metabolites together form a complex network that shapes the metabolic state of postoperative patients^[68]^. Future research should explore the interactions among these mechanisms and quantify their relative contributions to PIR, providing novel insights and strategies for its prevention and management.

## GUT MICROBIOTA INTERVENTION STRATEGIES

Gut microbiota intervention strategies show promising potential for alleviating PIR. The main approaches and their underlying mechanisms are outlined below.

### Probiotics and prebiotics

Probiotics and prebiotics are key strategies for modulating the gut microbiota. Studies suggest that they can lower the risk of PIR by increasing the abundance of beneficial bacteria and improving microbial composition and function^[69]^. Strains such as Lactobacillus and Bifidobacterium improve insulin sensitivity through multiple mechanisms. They metabolize dietary fiber to produce SCFAs such as acetate, propionate, and butyrate, which exert anti-inflammatory effects and regulate energy metabolism.

Additionally, probiotics inhibit the growth of pathogenic bacteria through competitive exclusion and the production of antimicrobial substances, thereby maintaining microbial balance. They also strengthen the intestinal barrier by promoting mucin secretion and the expression of tight junction proteins, which helps limit the translocation of bacterial endotoxins such as LPS^[70,71]^. Prebiotics such as inulin and fructooligosaccharides act as substrates for beneficial bacteria, supporting their growth and metabolism, and indirectly improving insulin sensitivity.

### Fecal microbiota transplantation

FMT involves transferring fecal material from healthy donors into the intestine of recipients to restore gut microbiota balance. Clinical studies indicate that FMT can markedly improve the metabolic status of patients with PIR. It has been shown to increase microbial diversity, restore beneficial taxa, reduce inflammation, and enhance insulin sensitivity. Nevertheless, FMT faces challenges in clinical applications, such as donor screening, standardization of procedures, and ethical considerations^[72,73]^. Rigorous donor screening is essential to exclude infectious diseases and other health risks. Standardized transplantation protocols will improve consistency and reproducibility of outcomes. In addition, the ethical issues surrounding FMT require careful evaluation and resolution.

### Dietary modulation

After surgery, once a normal diet is resumed, dietary management plays an important role in regulating the gut microbiota and mitigating PIR. A fiber-rich diet enhances SCFA production, strengthens the intestinal barrier, reduces inflammation, and improves insulin sensitivity. Studies have shown that Mediterranean and low-carbohydrate diets exert beneficial effects on the gut microbiota and PIR^[74,75]^. The Mediterranean diet, characterized by high consumption of fruits, vegetables, whole grains, nuts, and olive oil, has anti-inflammatory and antioxidant properties that support microbial health. Low-carbohydrate diets help reduce insulin resistance by limiting sugar intake. Conversely, excessive consumption of high-fat and high-sugar foods impairs gut microbial balance and increases PIR risk.

### Pharmacological interventions

Pharmacological agents can also modulate the gut microbiota to improve PIR. For example, metformin has been shown to increase the abundance of *Akkermansia muciniphila*, enhance barrier integrity, suppress inflammation, and improve insulin sensitivity^[76]^. Other agents, including probiotic preparations and anti-inflammatory drugs, also show potential. Probiotic preparations support microbial balance and insulin sensitivity, while anti-inflammatory drugs reduce cytokine production and thereby improve metabolic outcomes^[77,78]^.

Targeted modulation of the gut microbiota offers an effective means of alleviating PIR. Future research should focus on elucidating underlying mechanisms, verifying clinical efficacy, and developing innovative therapeutic approaches that expand the options for prevention and treatment.

## CONCLUSION

The gut microbiota is increasingly recognized as a key factor in PIR, providing new opportunities for prevention and treatment through targeted microbiota modulation. Although numerous studies have reported correlations between the gut microbiota and PIR, direct causal links remain incompletely understood. This article has several limitations. Much of the mechanical evidence comes from animal models, while clinical data remain limited. The causal dynamics of host-microbiota interactions have not been fully established. Future work should integrate metagenomics and metabolomics to identify PIR-specific microbial markers, develop engineered bacteria for targeted SCFA delivery, and advance perioperative microbiota monitoring technologies. Importantly, the safety and efficacy of these novel interventions must be validated in rigorous animal studies and clinical trials. In conclusion, the gut microbiota plays a central role in the onset and progression of PIR. Deeper mechanistic insights, coupled with the development of safe and effective interventions, will open new avenues for clinical management of PIR and related metabolic diseases.

## DECLARATIONS

### Copyright

© The Author(s) 2025.
